# Differential Expression Analysis of tRNA-Derived Small RNAs from Subcutaneous Adipose Tissue of Obese and Lean Pigs

**DOI:** 10.3390/ani12243561

**Published:** 2022-12-16

**Authors:** Hao Gu, Mailin Gan, Linghui Wang, Yiting Yang, Jinyong Wang, Lei Chen, Shunhua Zhang, Ye Zhao, Lili Niu, Dongmei Jiang, Yiwu Chen, Anan Jiang, Linyuan Shen, Li Zhu

**Affiliations:** 1 Department of Animal Science, College of Animal Science and Technology, Sichuan Agricultural University, Chengdu 611130, China; 2 Farm Animal Genetic Resource Exploration and Innovation Key Laboratory of Sichuan Province, Sichuan Agricultural University, Chengdu 611130, China; 3 Chongqing Academy of Animal Science, Chongqing 402460, China; 4 College of Animal Sciences, Jilin University, Changchun 130062, China

**Keywords:** tsRNA, obese, pig, subcutaneous adipose tissue, lipid deposition

## Abstract

**Simple Summary:**

Pig subcutaneous adipose tissue deposition capacity can reflect the growth rate of pigs, but too much fat deposition can lead to obesity, causing serious metabolic disorders, resulting in metabolic diseases. At present, obesity has become a global health epidemic. In this study, we used obese and lean pigs as models to reveal the expression profile of tsRNA in subcutaneous adipose tissue. The results demonstrated an important potential regulatory network involved in fat deposition in porcine subcutaneous adipose tissue. This study provides a new research idea for the regulation mechanism of fat deposition in pigs, and provides a reference for preventing the occurrence of obesity.

**Abstract:**

Epigenetic factors, including non-coding RNA regulation, play a vital role in the development of obesity and have been well researched. Transfer RNA-derived small RNA (tsRNA) is a class of non-coding RNA proven to be involved in various aspects of mammalian biology. Here we take pigs as a model for obesity research and use tsRNA-seq to investigate the difference in tsRNA expression in the subcutaneous adipose tissue of obese and lean pigs to elucidate the role of tsRNA in obesity development. A total of 482 tsRNAs were identified in pig adipose tissue, of which 123 were significantly differentially accumulated tsRNAs compared with the control group. The tRF-5c was the main type of these tsRNAs. The largest number of tsRNAs produced was the Gly-carrying tRNA, which produced 81 tsRNAs. Functional enrichment analysis revealed that differential tsRNAs indirectly participated in MAPK, AMPK, insulin resistance, the TNF signaling pathway, adipocytokine signaling pathway, and other signaling pathways by interacting with target genes. These are involved in bioenergetic metabolic regulatory processes, suggesting that tsRNAs may influence these pathways to mediate the regulation of energy metabolism in porcine adipocytes to promote lipid deposition, thus contributing to obesity. Our findings suggest a potential function of tsRNA in regulating obesity development.

## 1. Introduction

As one of the most widely raised livestock in the world, pigs not only provide more than 30% of the world’s protein [1] but are also an ideal animal model for human medicinal research [2]. Because of its similarity with humans in terms of body size, cardiovascular system structure, physiology, and metabolic characteristics [3], it has been widely used in the study of diseases. The incidence of obesity in humans is increasing [4,5] and can cause serious metabolic disorders such as type 2 diabetes [6,7], nonalcoholic fatty liver disease [7,8], hypertension, and cardiovascular diseases [9,10]. Therefore, it is of great significance to understand the molecular mechanisms of obesity.

Yorkshire pigs, known for their fast growth and a high percentage of lean meat, are intensively bred worldwide. QingYu pigs, on the other hand, are a local breed in China that have a low feed conversion rate and a strong fat deposition ability, and are considered a fat breed. In pig production, fat deposition is important since it is a key economic characteristic that affects the production efficiency of pigs. Studies have shown that the thickness of subcutaneous fat is negatively correlated with lean meat percentage and positively correlated with intramuscular fat content [11,12]. Therefore, the purpose of artificial breeding in the pig breeding industry is to reduce the thickness of subcutaneous fat to increase the lean meat percentage, enhance pork quality and reduce production costs.

Adipose tissue is important for thermoregulation, storing and providing energy, and protecting internal organs [13,14]. Adipogenesis is a complex process regulated by numerous transcription factors, secretory factors, hormones, and functional genes [15,16,17]. More and more studies have shown that non-coding RNA, including lncRNA [18,19], circRNA [20,21], miRNA [22,23], and tsRNA [24], play a key role in adipogenesis. Among them, tRNA-derived small RNAs (tsRNAs) are a relatively less studied class of non-coding RNAs. They are derived from tRNAs cleaved by specific nucleases, which were initially considered the waste products formed by transcription [25]. However, with the development of high-throughput sequencing technology and the maturity of bioinformatics analysis, tsRNA is gradually being considered small molecule RNA with important functions at the transcriptional and post-transcriptional levels, and for the regulation of translation by binding to ribosomes [26,27,28]. Previous studies have shown that tsRNA can be divided into two major types of tiRNA and tRFs according to the different cleavage sites on tRNA. Among them, tiRNA includes two subtypes (tiRNA-3, tiRNA-5), and tRFs include seven subtypes (tRF-1, tRF-2, tRF-3a, tRF-3b, tRF-5a, tRF-5b, tRF-5c) [29]. Depending on the cleavage site on the tRNA, the resulting tsRNA transcripts range in length from 14 to 50 nt. Studies on tsRNA revealed their role in cancer, neurological diseases, and inflammation [30,31]. Currently, only a few studies of tsRNA in obesity have been reported [24,32,33].

In this study, we used Yorkshire and QingYu pigs as research models to compare tsRNA gene accumulation profiles in the subcutaneous adipose tissue of two pig breeds, using high-throughput RNA-seq, functional enrichment, and interaction network analysis. We aimed to study the molecular mechanism of differentially expressed tsRNA regulating fat deposition as a novel approach to investigating the regulation of pig fat deposition. Furthermore, elucidating the molecular mechanism of fat deposition in pigs will not only benefit the genetic breeding of pigs, but also provide a theoretical basis for the study of human obesity and related metabolic diseases.

## 2. Materials and Methods

### 2.1. Experimental Animals

In this study, six unrelated Yorkshire and six unrelated QingYu female pigs spanning three generations were used. All animals were fed an equal-calorie diet, with free access to food and water. Animals were fasted the night before and slaughtered humanely when they reached a weight of 110 kg. All experimental animal studies were approved by the Animal Ethics and Welfare Committee of Sichuan Agricultural University, permission No. DKY-B20131403.

### 2.2. Sample Collection

Blood samples of obese and lean pigs were collected from the anterior vena cava immediately before being slaughtered. The blood was centrifuged at 3000× *g* for 15 min at 4 °C. The serum was collected and stored at −80 °C. After death, the subcutaneous fat thickness of the first rib, the last rib, and the last lumbar vertebra was measured using the vernier caliper [34]. Then the carcass was divided, the adipose tissue was separated and weighed, and the adipose tissue index (the percentage of adipose tissue weight vs. total carcass weight) was calculated. Subcutaneous adipose tissue was then preserved in liquid nitrogen for RNA extraction and tsRNA sequencing while 4% paraformaldehyde was used to preserve subcutaneous adipose tissue for histomorphometric analysis.

### 2.3. Determination of Serum Biochemical Indexes

The levels of total triglyceride (TG), total cholesterol (TC), high-density lipoprotein cholesterol (HDL), low-density lipoprotein cholesterol (LDL), and very low-density lipoprotein cholesterol (vLDL) in serum were detected using corresponding kits, which were provided by Nanjing Jiancheng Bioengineering Institute (Nanjing, China). Following the procedure from a previous study [35], the measurement of TG (#A110-1-1), TC (#A111-1-1), HDL (#A112-1-1), LDL (#A113-1-1), and vLDL (#H249) in serum was performed under different temperature and absorbance conditions according to the manufacturer’s instructions. The content of each index in the serum was calculated according to the standard sample.

### 2.4. RNA Extraction and RT-qPCR

According to the manufacturer’s instructions, RNA was extracted from subcutaneous fat tissue using TRIzol reagent (TaKaRa, Dalian, China). Reverse transcription was performed using the PrimeScript RT Master Mix kit (TaKaRa, Dalian, China) for mRNA, and the Mir-X miRNA First-Strand Synthesis Kit (TaKaRa, Dalian, China) for tsRNA. Real-time PCR of tsRNA and mRNA was performed using the TB Green Premix Ex Taq II Kit in a Bio-Rad CFX96 Real-Time PCR Detection System (Bio-Rad, Richmond, CA, USA). The expression levels of mRNA and tsRNA were normalized with *β-actin* and *U6* as controls, respectively. The relative expression levels of mRNA and tsRNA were calculated using the 2^−ΔΔCt^ method [36]. All primer sequences are listed in Supplementary Table S1.

### 2.5. tsRNA Sequencing

Sequencing of tsRNA was performed as previously stated [37]. Briefly, the quality of total RNA was detected using agarose gel electrophoresis and Nanodrop analysis. RNA samples were pretreated to remove RNA modifications that may interfere with the construction of the small RNA-seq library. The total RNA of each sample was sequentially ligated to 3′ and 5′ small RNA adapters. cDNA was then synthesized and amplified using Illumina’s proprietary RT primers and amplification primers. Subsequently, ~134–160 bp PCR amplified fragments were extracted and purified from the PAGE gel. Finally, the completed libraries were quantified in the Agilent 2100 Bioanalyzer. The libraries were denatured and diluted to a loading volume of 1.3 ml and loading concentration of 1.8 pM. Diluted libraries were loaded onto a reagent cartridge and forwarded to sequencing run on an Illumina NextSeq 500 system using the NextSeq 500/550 V2 kit (#FC-404-2005, Illumina San Diego, CA, USA), following the manufacturer’s instructions. The raw sequencing data was deposited in the National Genomics Data Center accession PRJNA899798.

### 2.6. tsRNA Analysis

The tsRNA sequencing data were analyzed as reported previously [38]. Low-quality reads and adapter sequences were removed from raw data using BOWTIE software. The tRNA sequences used to generate the tRNA libraries were obtained from GtRNAdb (http://gtrnadb.ucsc.edu/, accessed on 11 August 2022) and tRANscan-SE (http://lowelab.ucsc.edu/tRNAscan-SE/, accessed on 15 August 2022). The abundance of tsRNAs was evaluated using their sequencing counts and was normalized as counts per million (CPM) of total aligned reads. The tsRNAs differentially expressed were screened based on the count value with the R package edgeR [39].

### 2.7. Target Gene Prediction and Functional Enrichment Analysis

The target genes of tsRNA were predicted using the online website platform Omicstudio (https://www.omicstudio.cn/analysis, accessed on 8 September 2022). To obtain effective target genes, we took the intersection of the two according to the results on the Miranda and Targetscan databases. Next, we performed enrichment analysis on the genes in the intersection, and the signal pathways involved in tsRNA were revealed by Gene ontology (GO) and Kyoto Gene and Encyclopedia of Genomes (KEGG) enrichment analysis. Cytoscape software was used to construct a tsRNA-target gene-signaling pathway regulatory network. The predicted target genes are shown in Supplementary Table S2.

### 2.8. Statistical Analysis

Data were expressed as Mean ± SEM. Microsoft Excel and SPSS software version 26 (IBM, Armonk, NY, USA) were used to collate and analyze the data. A Student’s *t*-test was used to compare the data between the two groups. *p* < 0.05 was considered significant, and *p* < 0.01 was considered to be extremely significant.

## 3. Results

### 3.1. Phenotypic Differences between Obese and Lean Pigs

**Figure 1:**
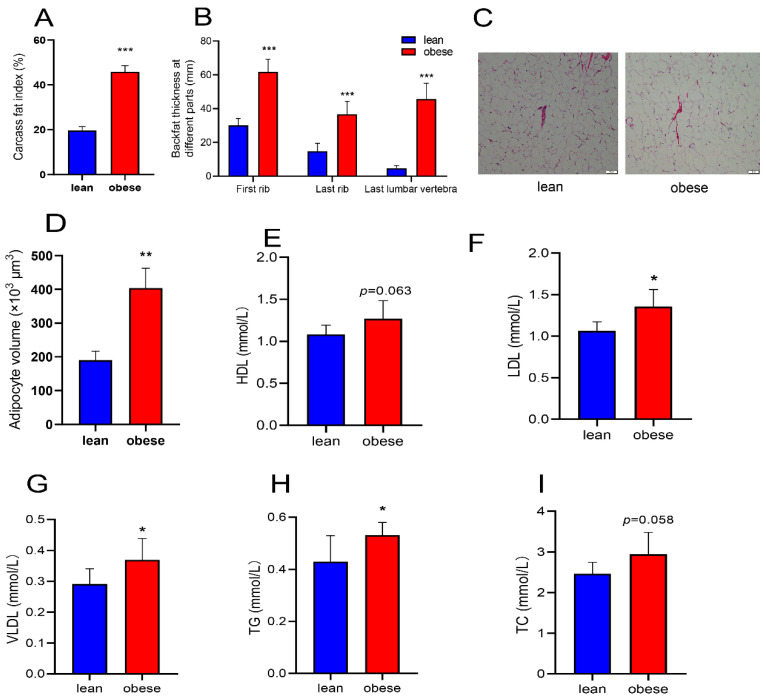
Phenotypic indexes of obese and lean pigs. (**A**) The percentage of adipose tissue in the carcass. (**B**) Subcutaneous fat thickness in different parts of the carcass. (**C**) Subcutaneous adipose tissue by HE staining. (**D**) Adipocyte volume; HDL (**E**), LDL (**F**), vLDL (**G**), TG (**H**), and TC (**I**) in the serum of obese and lean pigs. * *p* < 0.05, ** *p* < 0.01, *** *p* < 0.001.

In this study, we fed two different breeds of pigs that represented the obese and lean pig models. To prove that the models of obese and lean pigs were successfully established, the pigs were sacrificed and their subcutaneous fat was analyzed. The results showed that the fat index of the obese pigs, which was 2.33 times that of lean pigs, was significantly higher than that of the lean pigs (*p* < 0.01) (Figure 1A). By measuring the thickness of subcutaneous fat on different parts of the carcass, we found that the thickness of subcutaneous fat on the first rib, the last rib, and the last lumbar spine of obese pigs was 2.05, 2.47, and 9.78 times, respectively, that of the lean pigs. This difference was significant (*p* < 0.001) (Figure 1B). The HE staining technique was used to stain the subcutaneous fat. It can be seen that the size of subcutaneous fat cells in obese pigs was larger than that in lean pigs. The statistical results also showed that the volume of the fat cells in obese pigs was significantly larger than that in lean pigs (*p* < 0.01) (Figure 1C,D). Serum-related indicators of the two pigs suggested that the contents of high-density lipoprotein (HDL), low-density lipoprotein (LDL), and very low-density lipoprotein (vLDL) in the serum were elevated in obese pigs (Figure 1E–G). Both triglyceride (TG) and cholesterol (TC) also had the same trend (Figure 1H,I). Based on these results, it was clear that the fat content in obese pigs was higher than that in lean pigs, and the animal model was successfully established.

### 3.2. Accumulation Characteristics of tsRNA in Subcutaneous Fat

**Figure 2:**
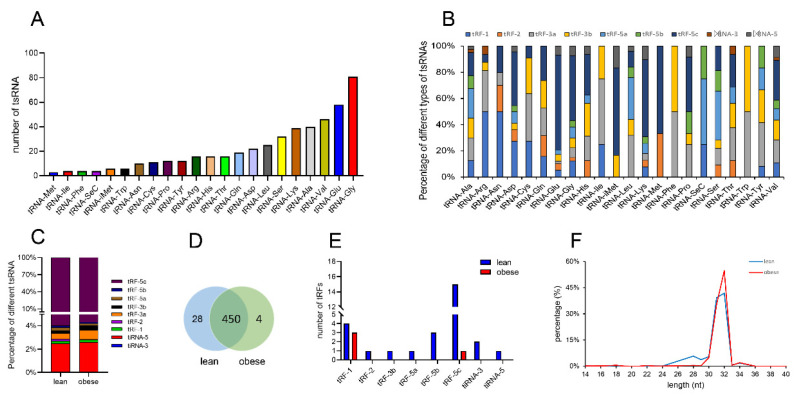
Characteristics of tsRNA in the subcutaneous adipose tissue of lean and obese pigs. (**A**) The number of tsRNAs derived from different tRNA isoforms. (**B**) Types of tsRNAs produced by tRNAs carrying different amino acids. (**C**) Different tsRNA types specific to obese and lean subcutaneous fat. (**D**) Venn diagram of tsRNA quantity in obese and lean subcutaneous fat. (**E**) Percentage of different types of tsRNA. (**F**) tsRNA length distribution.

Analysis showed that tsRNAs were derived from different tRNAs isoforms, with the least tsRNAs obtained from tRNA-Met and the most tsRNAs obtained from tRNA-Gly, which produced 3 and 81 tsRNAs, respectively (Figure 2A). The analysis of different types of tsRNA showed that tRNA-Met, tRNA-Phe, and tRNA-Trp produced the fewest tsRNA types, and all obtained 2 kinds of tsRNAs. The tRNA-Ala, tRNA-Gly, and tRNA-Val obtained the most tsRNA types, and 8 different tsRNAs were obtained (Figure 2B). A total of 482 tsRNAs were obtained from subcutaneous adipose tissue (Figure 2D). There was an overlap of 93.36% of the tsRNAs in lean and obese, with 28 and 4 tsRNAs unique to lean and obese, respectively, belonging to different types of tsRNAs (Figure 2E). Among the 9 different tsRNAs subtypes, tRF-5c was the most abundant type in both obese and lean pigs (Figure 2C). The length of these tsRNAs was in the range of 14–40 nt, of which 31 and 32 nt tsRNAs were the most abundant in lean and obese types, accounting for 81.09% and 90.05%, respectively (Figure 2F).

### 3.3. Difference Analysis of tsRNA in Subcutaneous Fat

**Figure 3:**
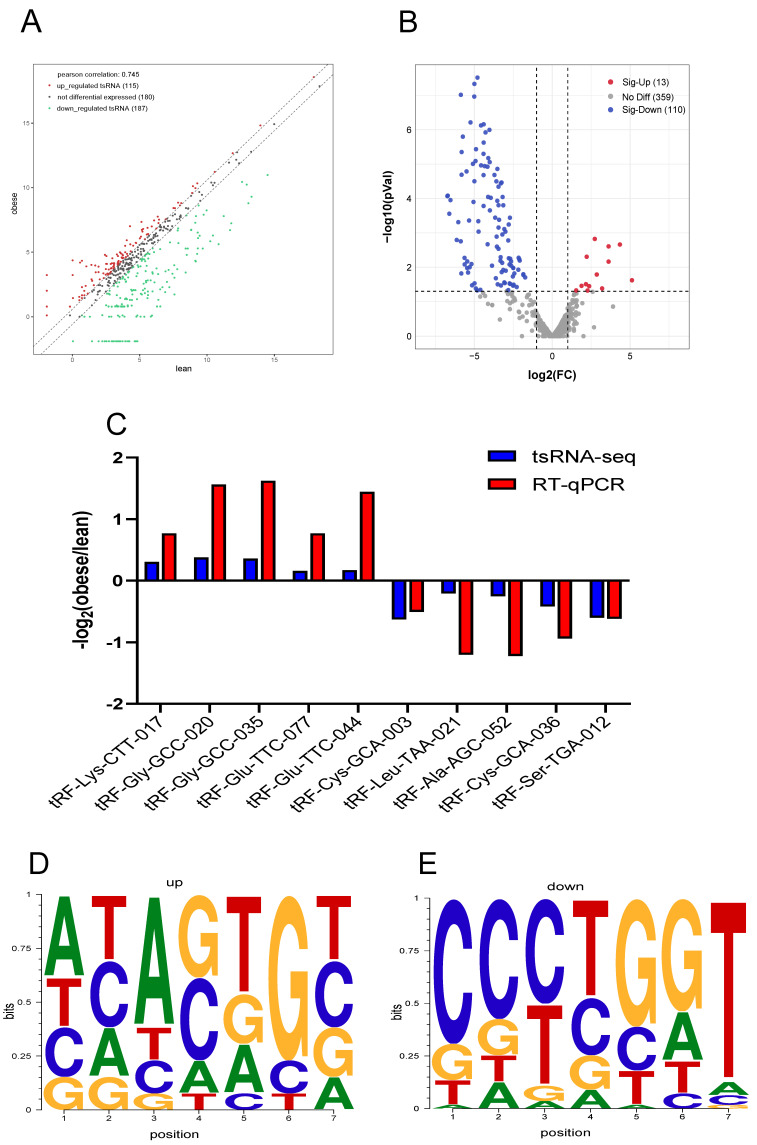
Analysis of differentially expressed tsRNAs. Scatter plot (**A**) and a volcano plot (**B**) of differentially expressed tsRNA, red dots represent increased tsRNA in obese pigs compared with lean pigs, blue dots represent decreased tsRNA in obese pigs compared with lean pigs. (**C**) RT-qPCR verified tsRNA sequencing results (*n* = 6). (**D**,**E**) Bias of differentially expressed tsRNA seed sequences.

Through the differential analysis of the accumulation of tsRNA in the subcutaneous fat of obese and lean pigs, we found that 115 RNAs were up-regulated and 187 were down-regulated in obese pigs (Figure 3A). Subsequently, tsRNAs with a Fold change ≥ 1.5 and *p* < 0.05 were selected as differentially expressed. Through differential analysis, a total of 123 differentially expressed tsRNAs were identified. Among them, 13 were significantly up-regulated and 110 were significantly down-regulated in obese pigs (Figure 3B). To verify the accuracy of the sequencing data, tsRNAs from several differential tables were randomly selected and verified by RT-qPCR. The results showed that the tsRNAs identified by the two methods had the same accumulation trend in the two different porcine subcutaneous adipose tissues (Figure 3C). Finally, we analyzed the base preference of the seed sequence of the differentially expressed tsRNA (Figure 3D,E), and the results indicated that the differentially expressed tsRNA seed sequence had different preferences, which also implied that the way in which it works was different.

### 3.4. Target Gene Prediction and Functional Analysis of Differential tsRNAs

**Figure 4:**
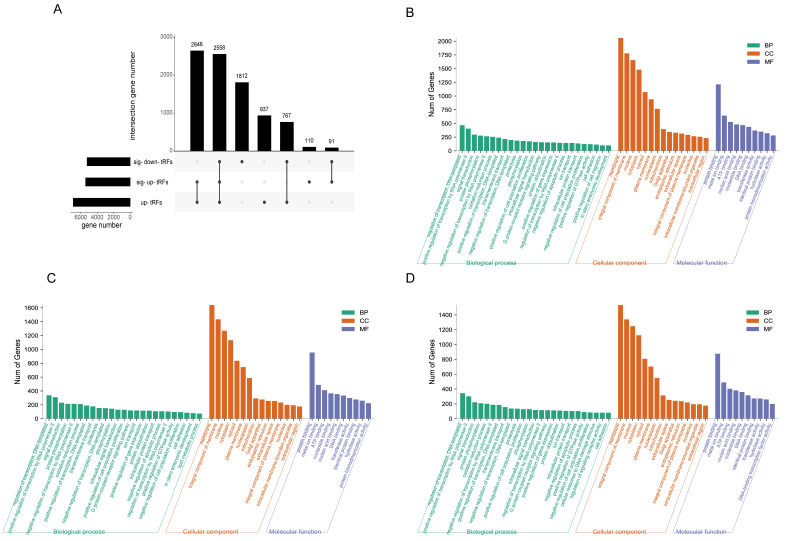
tsRNA target genes prediction and GO enrichment analysis. (**A**) The number of target genes and gene intersection distribution; GO enrichment analysis of target genes of increased (**B**), significantly increased (**C**), and significantly decreased (**D**) tsRNAs.

**Figure 5:**
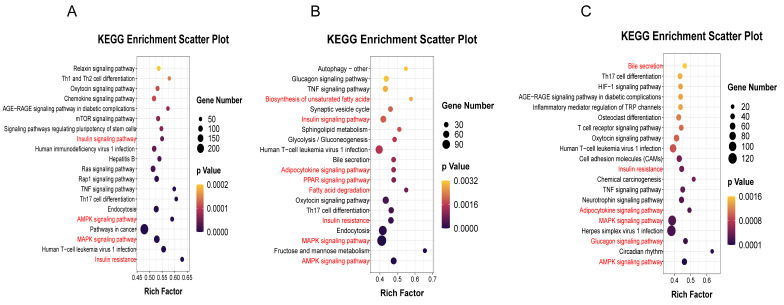
KEGG enrichment analysis of tsRNA target genes. Increased (**A**), significantly increased (**B**), and significantly decreased (**C**) tsRNA target gene KEGG signaling pathway enrichment analysis.

Studies have shown that tsRNA has a similar function to miRNA, which regulates its expression by binding to target genes through seed sites [40]. Here, we predicted the top five tsRNA target genes with significant differential expression. Since the up-regulated but not statistically significant tsRNA also play an important role in obesity, we also included the up-regulated top 10 tsRNA in the analysis. As shown in Figure 4A, the three categories each have a similar number of target genes and 2558 target genes are common. The up-regulated and differentially up-regulated tsRNAs have 2646 target genes in total. GO analysis (Figure 4B–D) showed that the differentially upregulated tsRNAs are involved in lipid metabolism and redox process in biological processes (Figure 4C). The results of the KEGG enrichment analysis showed that all of them were enriched in MAPK, AMPK, and insulin resistance signaling pathways (Figure 5A–C). Up-regulated and significantly up-regulated tsRNAs were also enriched in the insulin signaling pathway (Figure 5A,B), and significantly up-regulated tsRNAs were also enriched in pathways related to fat and fatty acid metabolism.

### 3.5. Regulation Network and Correlation Analysis of Fat Metabolism-Related Pathways

**Figure 6:**
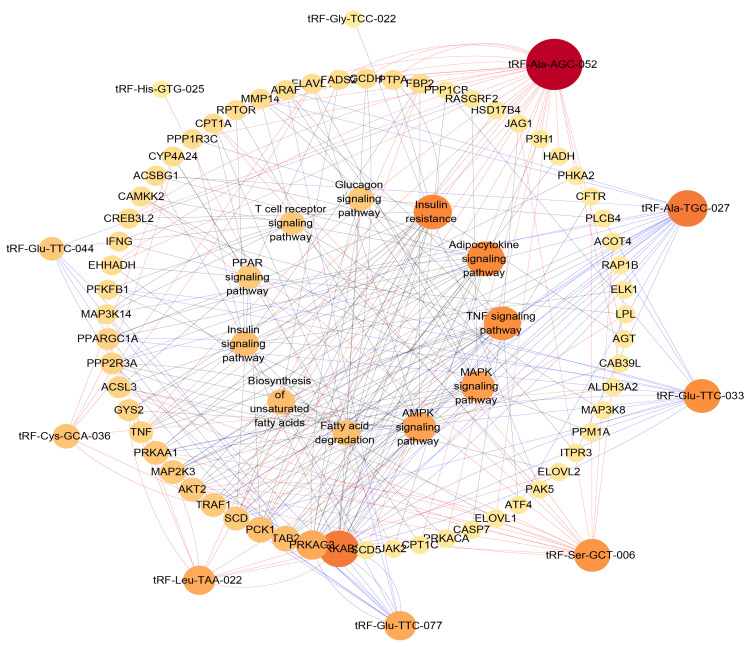
tsRNA-target gene-pathways network interactions. Diagram of the regulation network of tsRNA-target gene-fat development-related pathways. The red line indicates differential upregulation of tsRNA, the blue line indicates differential downregulation.

**Figure 7:**
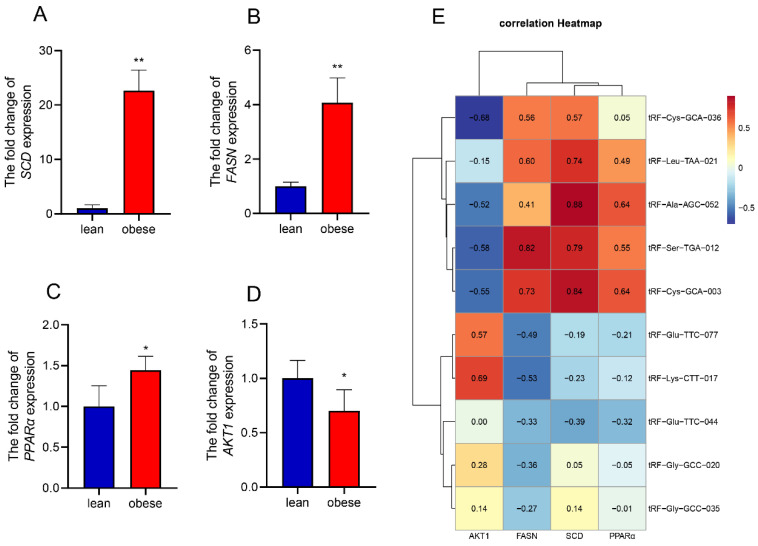
tsRNA regulatory network and correlation analysis. (**A**–**D**) Detection of relevant gene expression levels by RT-qPCR (*n* = 6). (**E**) Heat map of correlation between tsRNA and selected genes in metabolic pathways. * *p* < 0.05, ** *p* < 0.01.

Here, we first constructed an interaction network diagram of tsRNA-target gene–fat development-related pathways to further illustrate the important role of tsRNA in pig fat deposition. In the interaction network diagram, tRF-Ala-AGC-052 and tRF-Ala-TGC-027 targeted the most target genes in the upregulated and downregulated tsRNAs, respectively, and participated in many signaling pathways related to lipid metabolism (Figure 6). Next, we detected the expression levels of related genes in these metabolic pathways using RT-qPCR in the subcutaneous adipose tissues of obese and lean pigs. Then, we selected some genes involved in important components of the network map from these pathways for verification, we found that the FASN, SCD, and PPARα genes were upregulated in obese pigs and AKT1 was downregulated in obese pigs (Figure 7A–D). Correlation analysis of tsRNA and gene expression revealed that they have the same expression trend by correlation heat map (Figure 7E).

## 4. Discussion

Epigenetics is involved in the whole life cycle of an organism from embryonic development to death. It affects every microscopic change in the body and thus affects the phenotypic changes as well [41]. Non-coding RNAs are involved in the regulation of gene expression in a variety of different ways [42]. tsRNA is a new type of non-coding small molecule RNA. It is highly conservative and participates in many biological processes [29]. Studies have shown that tsRNA is involved in adipocyte differentiation and it plays a critical role in adipose development [33,43,44].

Energy metabolism is a fundamental feature of life. In mammals, the liver, muscle, and fat are important metabolic centers that play a vital role in energy metabolism and internal environmental homeostasis [45,46]. When the body ingests too much energy, the excess energy is stored in the form of fat. Adipose tissue has high plasticity, and with the continuous accumulation of energy, the phenotype of adipocytes will change. For example, the volume of adipocytes will expand exponentially and the type of metabolism will change, causing insulin resistance [47,48,49].

As an ideal experimental animal model, pigs are widely used in metabolic disease studies of obesity and diabetes [3,50]. However, the fat content of pigs has also been studied as an important economic trait in animal husbandry [51], and its fat deposition ability will affect pig carcass quality, lean meat rate, and reproductive performance [52,53,54]. Therefore, subcutaneous fat thickness is a key indicator for assessing fat deposition in pigs and the reproductive performance of sows, and is regarded as a significant trait in pig breeding. In this study, we used pigs as animal models to explore the effect of tsRNA in subcutaneous fat on the fat deposition ability of obese and lean pigs, which will contribute to a new understanding of adipogenesis.

We used two different pig breeds, obese (QingYu pigs) and lean (Yorkshire pigs) for our study. The differences in fat deposition ability between the two breeds were illustrated in terms of carcass fat content and subcutaneous fat thickness. The HE staining analysis of subcutaneous adipose tissue revealed that obese pigs had a larger adipocyte volume and triglyceride content compared to lean pigs, which led to increased obesity [55]. Additionally, we found increased levels of cholesterol, low-density lipoprotein, and other biochemical indicators in the serum of obese pigs. A large number of studies have shown that increased levels of triglycerides and cholesterol in the body will exacerbate obesity and its resulting metabolic syndrome [56,57,58].

By sequencing the tsRNA in obese and lean subcutaneous pig fat, we found that the length of these tsRNAs was mainly 31–32 nucleotides, and the type was mainly tRF-5c, indicating that tsRNA was not produced by the random degradation of tRNA as previously thought [44]. A large number of studies have shown that tsRNA is involved in many aspects and plays an important functional role. For example, Jin et al. [37] identified novel tsRNAs in human and mouse pancreatic cancer tissues and serum using RNA sequencing and in situ hybridization, and proved that tRF-Pro-AGG-004 and tRF-Leu-CAG-002 can be used as new biomarkers for the early diagnosis of pancreatic cancer. The deletion of tRNA RNase (Angiogenin) in mice leads to a decrease in tsRNA production in sperm, which induces paternal inflammation and heritable metabolic disorders [59]. TsRNA-16902 regulates adipogenic differentiation of human bone marrow mesenchymal stem cells by targeting *PARγ* and participating in the smad2/3 signaling pathway [24]. Chen et al. [60] demonstrated that metabolic disorders in offspring are due to changes in tsRNAs in sperm caused by the paternal high-fat diet, which is inherited and causes metabolic disorders in offspring.

To further understand the function of tsRNA, we predicted and functionally enriched the target genes of differential tsRNA. GO analysis showed that the differentially upregulated tsRNA in obese pigs was specifically enriched in lipid metabolism, redox processes of a large number of genes, and ion transport processes, which was consistent with a high-fat content phenotype in obese individuals [61]. KEGG pathway enrichment analysis showed that the differentially expressed tsRNAs were mainly involved in the MAPK, AMPK, insulin resistance, TNF signaling, and adipocytokine signaling pathways. It is worth noting that the differentially upregulated tsRNAs were also involved in the PPAR signaling pathway, fatty acid metabolism, and other signaling pathways related to obesity and fat deposition, which can promote the development of adipocytes and lipid accumulation [62,63]. The differentially downregulated tsRNAs were involved in the Glucagon signaling pathway and bile secretion.

The results of our analysis proved that tsRNA is involved in fat deposition in pigs and plays a key role in the process. By constructing the regulatory network diagram, it can be seen that tRF-Ala-AGC-052, tRF-Ser-GCT-006, tRF-Ala-TGC-027, and tRF-Glu-TCC-033 are the most important factors in differential tsRNA, and function in the AMPK, MAPK, insulin resistance, and adipocytokine signaling pathways, which are inextricably linked to lipid metabolism [64,65]. The process of obesity is accompanied by the dynamic expression of a large number of genes [66]. We found that the expression levels of genes related to fatty acid metabolism, fat development, and insulin resistance in the above pathways were different in obese and lean pig subcutaneous fat [67,68]. This suggests that the functional intensity of these signaling pathways was different, which may influence fat deposition. Finally, we analyzed the correlation between tsRNA and the expression of these genes and found that they had the same expression pattern. However, to further explore the specific function of tsRNA in adipocyte development, a large number of functional verification experiments are needed.

## 5. Conclusions

In conclusion, we revealed the accumulation profile of tsRNA in subcutaneous adipose tissue of obese and lean pigs and screened 123 differentially expressed tsRNAs. The function of some tsRNAs was predicted, and functional enrichment analysis showed that the differential tsRNAs were involved in signaling pathways related to fat metabolism. Our study is conducive to a better understanding of the role of tsRNA in fat metabolism and deposition and provides a theoretical basis for the molecular genetics and breeding of pigs.

## Data Availability

If additional data related to this study are required, please consult the corresponding authors.

## Supplementary Materials

The following supporting information can be downloaded at: https://www.mdpi.com/article/10.3390/ani12243561/s1. Table S1: Sequences of RT-qPCR primers used in this study; Table S2: Target genes prediction of tsRNAs.
